# Subcutaneous Terbutaline Pump for Maintenance of Tocolysis following Arrest of Acute Preterm Labor

**DOI:** 10.1155/2014/795329

**Published:** 2014-03-31

**Authors:** Kavita Singh, Mohammed T. Ansari, Laura M. Gaudet

**Affiliations:** ^1^Clinical Epidemiology Program, the Ottawa Hospital Research Institute, P.O. Box 201B, TOH-General Campus, 501 Smyth Rd, Ottawa, ON, Canada K1H 8L6; ^2^Maternal Fetal Medicine, Horizon Health Network, Room 2510, 135 MacBeath Avenue, Moncton, NB, Canada E1C 6Z8

Drs. Elliott and Morrison recently published a narrative review about the effectiveness of oral and subcutaneous maintenance of tocolytic agents following successful arrest of acute preterm labor [1]. The authors conclude that subcutaneous terbutaline pump is beneficial and safe for maintenance of tocolysis based on all the available evidence.

While the authors have provided an exhaustive list of the 46 published studies that have examined the terbutaline pump, we would like to express some concerns about the interpretations of this evidence. The authors have formed conclusions without assessing study quality or synthesizing results. In addition, several of the studies cited are single-arm case series and we would be hesitant to draw any conclusions about the efficacy based on this weak study design.

We were commissioned by the Agency for Healthcare Research and Quality to evaluate the benefits and harms of subcutaneous terbutaline pump for maintenance of tocolysis by conducting a systematic review of the literature [2, 3]. Using prespecified eligibility criteria, we included 14 unique studies [3–17]. The evidence base that we relied on to form our conclusions included all randomized and comparative observational studies. These studies have been cited by Drs. Elliott and Morrison. Of the remaining 33 studies cited by the authors, we had identified and excluded 29; primarily because these studies did not include a comparator group (i.e., they were single-arm studies). The 4 studies that were not identified by our search [18–21] would also have been excluded because there were no nonpump controls.

These 14 studies were of lower quality (medium to high risk of bias) and several studies examined data from one proprietary database. Although we found that the pump was beneficial for some outcomes (i.e., neonatal death, incidence of delivery <32 weeks and <37 weeks, and prolonging pregnancy), importantly, these benefits were rated as having low strength of evidence, which means that we have low confidence that the evidence reflects the true effect [22]. The strength of evidence is a subjective—though systematic and transparent—assessment of the reviewers' confidence in the findings based on the overall risk of bias, inconsistency, indirectness, and imprecision of the body of evidence [22]. In addition, there was insufficient data on other clinically important outcomes, such as bronchopulmonary dysplasia, intraventricular hemorrhage, and neonatal and maternal harms.

Our more cautious interpretation about the efficacy and safety of terbutaline pump is based on a systematic review of the evidence and scientific methods for quantitative and qualitative synthesis (i.e., meta-analysis and grading strength of evidence). While we acknowledge that the harms reported by FDA after marketing surveillance do not establish causality [23], we have shown that the evidence for efficacy has limited validity. As such, we maintain that the safety and efficacy of the pump for maintenance tocolysis are unclear. Its use should be limited to the research setting, such as further investigation in an adequately powered, randomized, controlled trial.
